# Travelling Far and Wide? Applying the Multiple Streams Framework to Policy-Making in Autocracies

**DOI:** 10.1007/s11615-022-00393-8

**Published:** 2022-04-22

**Authors:** Nicole Herweg, Nikolaos Zahariadis, Reimut Zohlnhöfer

**Affiliations:** 1 Institut für Politische Wissenschaft, Ruprecht-Karls-Universität, Bergheimer Straße 58, 69115 Heidelberg, Germany; 2 Rhodes College, 2000 N. Parkway, 38112 Memphis, TN USA

**Keywords:** Agenda-setting, Decision-making, Problem definition, Nondemocratic regimes, Policy change, Agenda-Setting, Entscheidungsfindung, Multiple-Streams-Ansatz, Problemdefinition, Politikwandel

## Abstract

The Multiple Streams Framework (MSF) builds on the concepts of timing and ambiguity and their effects on the policy process. Since its introduction to agenda-setting in the U.S. presidential system, scholars have transferred the MSF’s core ideas to multiple issue areas, policy stages, and political systems. However, what has been lacking so far is a thorough discussion of the MSF’s travelling capacity to nondemocratic forms of government. Building on a brief summary of the MSF’s main ideas, this article discusses the challenges that policy-making in autocracies poses for MSF applications and ways to adapt it to the peculiarities that are typical for these regimes. The article focuses on the agenda-setting stage in which formal institutions are less important and introduces falsifiable hypotheses explaining agenda change. Due to tremendous differences regarding the organization of the decision-making process in autocratic regimes, the article only sketches out how the MSF could be adapted to explain policy change in this institutional setting. The article concludes with a discussion of whether the MSF is stretched too far by applying it to nondemocratic systems. It turns out that in theoretical and conceptual terms, the MSF travels surprisingly well to these systems.

## Abstract (de)

Der Multiple-Streams-Ansatz (MSA) basiert auf den Konzepten Zeitknappheit und Ambiguität und ihrer Bedeutung für den politischen Prozess. Seit der MSA für die Analyse des Agenda-Settings in den USA entwickelt wurde, sind seine Kerninhalte auf eine Vielzahl von Politikfeldern, Phasen des Policy-Zyklus und politische Systeme übertragen worden. Was bislang allerdings noch weitgehend fehlt, ist eine Diskussion der Frage, inwieweit der Ansatz sich auch auf Nichtdemokratien übertragen lässt. Nach einer kurzen Zusammenfassung des MSA diskutiert dieser Aufsatz die Herausforderungen einer Übertragung des Ansatzes auf Autokratien und präsentiert Möglichkeiten, ihn an die Besonderheiten dieser politischen Systeme anzupassen. Wir konzentrieren uns auf die Agenda-Setting-Phase, in der formale Institutionen eine geringere Rolle spielen, und schlagen falsifizierbare Hypothesen vor, mit denen Agendawandel erklärt werden kann. Aufgrund der größeren Varianz hinsichtlich der Institutionen der Entscheidungsfindung in Autokratien skizzieren wir dagegen lediglich, wie der MSA auch Politikwandel in Nichtdemokratien erklären könnte. Abschließend diskutieren wir, ob der MSA bei der Anwendung auf Autokratien zu weit gedehnt wird. Tatsächlich finden wir aber, dass er sich zumindest auf der theoretischen und konzeptionellen Ebene erstaunlich gut auf solche politischen Systeme übertragen lässt.

## Introduction

Building on the concepts of timing and ambiguity, the Multiple Streams Framework (MSF) has been hugely successful academically, at least in terms of the number of empirical applications. Literature reviews find hundreds of applications in all different kinds of settings (Jones et al. 2016; Rawat and Morris 2016). What is more, in the last decade, the framework has also thrived conceptually, and a lot of theoretical refinement of the MSF has taken place (see Herweg et al. 2018 as an overview). For example, scholars have discussed how to apply the MSF to parliamentary systems (Herweg et al. 2015), to the particularities of policy-making in Latin America (Sanjurjo 2020), and even to the European Union (Herweg 2017; Zahariadis 2008). Similarly, the applicability of the MSF to stages other than agenda-setting has been investigated, focusing on decision-making (cf. Herweg et al. 2015; Howlett et al. 2015; Zohlnhöfer et al. 2016) but also taking into account policy implementation (Sager and Thomann 2017; Zahariadis and Exadaktylos 2016). Finally, and most recently, methodological issues regarding the MSF have also been considered in more depth (Engler and Herweg 2019; Zohlnhöfer et al. 2022).

What has been lacking so far, however, is a thorough discussion of the MSF’s travelling capacity to nondemocratic forms of government, which we group together under the label of autocracies. While Herweg et al. (2018, p. 45) reason that it should be possible, at least in principle, to apply the MSF in autocracies and mention some relevant needs for adaptation, they do not go into any details. Similarly, the recent literature reviews (Jones et al. 2016; Rawat and Morris 2016) do not touch upon this point at all. Finally, the papers that explicitly apply the MSF to autocratic settings either do not adapt the framework to their cases at all (Jafari et al. 2017), only discuss the need for adaptation in relation to the case they deal with (Ge et al. 2020; Zhu 2008), or only suggest possible adaptations inductively after the empirical analysis (Liu and Jayakar 2012). Exceptions are recent papers by Ararat Babayan et al. (2021) and Annemieke van den Dool (2022). In the former paper, the authors use existing MSF hypotheses to formulate expectations for their case study on Belarus, while van den Dool adapts some of the hypotheses suggested by Herweg, Zahariadis, and Zohlnhöfer (2018) to the Chinese case. A more general and systematic conceptual and theoretical assessment about how the framework could be applied in nondemocracies and how it might need to be adapted is still lacking in the policy process literature.

This is an unfortunate state of affairs because the differences between liberal democracies and nondemocratic regimes are substantial and are likely to matter a great deal for the applicability of the MSF. Certainly, autocrats must learn about problems, too, and solutions also have to be developed in autocracies; most importantly, autocracies also need to couple problems to policies, and the politics must be right for the adoption of a specific policy at that specific point in time. Nonetheless, these processes might play out very differently in democracies and nondemocracies. In the absence of media freedom, problem definition might not occur in public, and the government might even be able to ignore a major focusing event by censoring information and suppressing problem brokers. Similarly, many experts might not have access to the policy communities, and the technical feasibility of a proposed solution might be less relevant. Finally, changes of government are comparatively rare in autocracies, and the national mood might be irrelevant because of lack of media freedom, the possibility of suppressing opposition, and the absence of or severe restrictions on elections. Hence, the question is this: Can the MSF really travel to autocratic political regimes and improve our understanding of policy processes in nondemocracies that are so different from the political systems for which the MSF has been developed?

We define autocratic systems by their lack of contested elections in the sense of Alvarez et al. (1996) and Przeworski et al. (2000) (i.e., ex ante uncertainty, ex post irreversibility, and repeatability). Typically, in these systems we also find media censorship, restricted societal pluralism, and centralized political authority (Jones et al. 2019, p. 10; van den Dool 2022, p. 3). Of course, there are different types of autocratic regimes (Cheibub et al. 2010; Geddes et al. 2014; Wahman et al. 2013), and these differences are likely to have a substantial impact on the policy processes. Nonetheless, in this paper we try to make a more general argument about how the MSF could be adapted to characteristics of nondemocratic regimes that, hopefully, can be substantiated in more detail in future applications to specific regimes. In addition, while we are aware of the fact that in most autocracies it is not a single individual who makes all relevant decisions but rather a group with restricted access of varying size (depending on the type of regime), for simplicity’s sake we sometimes refer to “the autocrat” or “the dictator” to name the autocratic decision-making body (as Bueno de Mesquita et al. 2003 also do, for example).

In addition to filling an important research gap in the MSF literature by systematically discussing the need for adaptation when investigating autocratic regimes, this article also seeks to add to the literature on nondemocratic regimes. The literature on autocracies has suggested that applying “seminal theories of democratic politics (…) to authoritarian contexts” (Williamson and Magaloni 2020, p. 1539) is a promising avenue for analysing nondemocratic policy processes. One such seminal theory is the MSF, and we hope that this perspective will also provide new insights for research on autocracies.

We start by outlining the main ideas of the MSF. Next, we discuss the challenges policy-making in autocracies might pose for the application of the MSF and the ways in which the MSF could be adapted to cope with these peculiarities. We do this separately for MSF’s basic assumptions and its main elements, namely the three streams and the policy window. For the main elements, we derive a set of falsifiable hypotheses. We focus on the agenda-setting stage, in which formal institutions are less important (Baumgartner et al. 2009; Zohlnhöfer et al. 2016; Gandhi et al. 2020) and only sketch out how the MSF could be adapted to explain decision-making in these regimes (for China, see also van den Dool 2022, p. 7). The final section concludes by discussing whether the MSF is stretched too far by applying it to nondemocratic systems.

## The Multiple Streams Framework

The MSF is based on John Kingdon’s (1984) seminal book *Agendas, Alternatives, and Public Policies*, first published in 1984 (the following draws on Herweg et al. 2018). That book’s root idea is that policy-making is not necessarily an exercise in rational problem solving. Rather, certain actors from outside or inside of government—Kingdon calls them “policy entrepreneurs”—develop policy ideas, more or less independently of current problems, and try to couple their proposals to current problems at favourable points in time, which Kingdon calls “policy windows” (and which are sometimes labelled “windows of opportunity” in the literature). Hence, according to MSF thinking, policy-making resembles solutions looking for suitable problems rather than the other way around.

The framework rests upon some important assumptions, many of which were taken from the garbage-can model of organisational choice (Cohen et al. 1972). For example, policy-makers are assumed to have problematic (policy) preferences. Hence, most policy-makers do not really know what kinds of policies they prefer early in a policy process but tend to develop these preferences as the process progresses. This implies that policy entrepreneurs can manipulate policy-makers’ policy preferences at least to some extent. Manipulation—for example, framing a policy in a specific way—might make policy-makers more receptive to the policy proposals that policy entrepreneurs have worked out, which should facilitate getting these proposals on the agenda.

With regard to preferences, it has to be mentioned that policy entrepreneurs are not expected to act more rationally than policy-makers do. In particular, they might not have come up with the project they promote as a result of a rational selection process. Instead, they could just have stumbled over it and may have decided to lobby for it for all different kinds of reasons (including career motivations). Moreover, the assumption that policy-makers lack clear policy preferences does not exclude their having a clear preference for remaining in power, for example (Herweg et al. 2015, p. 437).

Another critical assumption of the MSF is that policy-makers have to decide under high levels of ambiguity and time constraints. Ambiguity is commonly defined as “a state of having many ways of thinking about the same circumstances or phenomena” (Feldman 1989, p. 5). Therefore, one can think of the COVID-19 crisis, for example, as a public health issue and also as a civil rights issue, an economic policy issue, or an issue regarding globalisation. In contrast to uncertainty, which can be reduced by additional information, ambiguity does not decrease with more data.

Moreover, policy-makers have next to no time to think through policy problems and potential solutions to them. Apart from the frequent need to respond quickly to a problem, these time constraints originate from the many issues that policy-makers need to attend to, which leaves extremely limited time to consider each individual problem thoroughly. Vice versa, policy-makers’ limited capacity to attend to problems also leads to competition between issues (and between the policy entrepreneurs who promote them) for policy-makers’ attention.

In addition, there is no comprehensive understanding of the entire policy process on the part of the participants of these very processes (“unclear technology”) while policy-makers flow in and out at various stages of the process (“fluid participation”).

According to MSF, three streams flow through the political system. The streams are assumed to be independent of each other; that is, developments in one stream do not affect the other two streams. The problem stream is about conditions that can turn into problems, which the political system then may have to deal with. Changes in specific indicators (unemployment rate, emissions, crime rate, etc.), focusing events such as severe accidents or crises, and feedback from existing programs all point to conditions that could become problems. Because problems are not considered evident facts in MSF thinking but are thought of as socially constructed, it depends to some extent on so-called problem brokers whether a condition can be framed as a problem. Knaggård (2015, p. 452) defines problem brokers as actors who “frame conditions as public problems and work to make policymakers accept these frames. Problem brokers thus define conditions as problems.” A problem broker can be, but does not need to be, identical to a policy entrepreneur.

In the policy stream, experts on specific issue areas develop policy alternatives. These experts propose, discuss, modify, and combine ideas in policy communities. At the end of a “softening-up” process, one or more worked-out alternatives become available that can rise to the political agenda. Viable alternatives are likely to fulfil certain “criteria for survival.” Policy alternatives that are technically feasible, that are affordable, that reflect the values of the policy community, and that are likely to find a majority in the political stream stand a better chance of being considered as viable policy alternatives and ultimately making it to the political agenda than proposals that do not fulfil these criteria.

Finally, the political stream deals with the political forces that can facilitate or impede an issue’s rise to the political agenda (and, finally, policy adoption). Core elements of the political stream are governments and parliaments (and their partisan composition), parties, interest groups, and also what Kingdon (1984) calls the “national mood,” that is, the perceived state of public opinion. The more favourable these core elements are towards a reform proposal (e.g., a majority in parliament whose ideological position is in line with the proposal, with little resistance or even support from interest groups), the more likely it is that the proposal will rise to agenda prominence and eventually be adopted.

For an agenda change to occur, all three streams need to be ready for coupling. That is to say, policy-makers need to perceive a problem that deserves attention (problem stream); a viable policy alternative (preferably one that meets the criteria for survival) needs to be available (policy stream); and at least one political entrepreneur—i.e., an actor who holds an elected leadership position, such as the relevant minister—must actively support the idea in question and must be willing to try to bring together a majority for it. But even if all three streams are ready for coupling, an agenda change is not necessarily forthcoming, according to the MSF. Rather, the coupling of the three independent streams becomes more likely at specific moments in time, the policy windows. Policy windows can open either in the problem stream or in the political stream (but not in the policy stream). A “problem window” can open when an indicator worsens substantially or when a crisis or feedback focuses attention on a specific problem. If the problem becomes to be perceived as threatening the government’s reelection, the government is very likely to act (Herweg et al. 2015, p. 437), and a policy window thus opens. A change of government or a swing in the (perceived) national mood may open a “political window” instead. When a policy window opens, a policy entrepreneur can try to couple her or his favourite proposal to the problem (in the case of a problem window) or can argue that her or his pet proposal fits nicely with the new government’s programmatic position or the current national mood. If this is successful, the policy entrepreneur’s pet proposal rises to the decision agenda.

While Kingdon’s original contribution was confined to the agenda-setting phase, more recent contributions have expanded the MSF to decision-making (see Herweg et al. 2018). One suggestion is to conceptualise successful agenda-setting as opening a “decision window.” During the process of decision coupling, which starts when the decision window opens, a political entrepreneur tries to stitch together the parliamentary majorities that are required for adoption of the project (Herweg et al. 2015). The political entrepreneur can make use of instruments such as package deals, concessions, and manipulation to get the proposal passed (Zohlnhöfer et al. 2016).

## Applying the MSF in Autocratic Regimes

In the following sections, we discuss how nondemocratic systems could differ from democracies in ways that are relevant for application of the MSF. We start with the framework’s basic assumptions before we discuss its core elements.

### Assumptions

There is little reason to expect that the MSF’s basic assumptions do not hold in autocratic regimes. Regarding unclear preferences, autocratic leaders as the main policy-makers are certainly interested in staying in office, but just as certainly they do not know exactly which kinds of policies they prefer in a given situation. Clearly, in a number of authoritarian regimes, a specific ideology dominates. While that may make specific policies less plausible in such systems, many proposals can be framed as fitting with the same ideology, so even if an ideology must be adhered to in an autocracy, that still leaves a lot of room for very different policies. Moreover, autocratic systems tend to be highly centralised (Babayan et al. 2021, p. 4; Jones et al. 2019). Consequently, a small group of leaders has to decide on all policies, which does not give them a reasonable chance to think through all options, given time and cognitive constraints and ambiguity, which should not be confined to democracies, either. Therefore, unclear preferences and the potential for manipulation become even more likely.

Nevertheless, one could question the assumption of fluid participation, but even that is true in large bureaucracies. Many decisions will also be prepared by lower-level bureaucrats, leaving them considerable discretion to address issues depending on their schedule and jurisdictional authority.

In contrast, the assumption of stream independence has been questioned for autocratic regimes (Liu and Jayakar 2012, p. 24; He and Li 2021, p. 161). The centralisation of the political system allows the autocrat to control the political stream, while he or she can keep a check on the problem stream via media censorship. Moreover, the policy stream is substantially impaired by the importance of the leader’s approval as the dominating criterion (see below). Hence, it is possible that streams flow less independently through the autocratic political system than assumed. Yet stream independence has been questioned for democracies, too, and we suggest treating stream independence in autocracies in the same way it is dealt with in analyses of democracies: as a conceptual device. Streams don’t necessarily have to be independent in all empirical cases—they only need to flow as if they are independent (Herweg et al. 2018, p. 39–40)!

### The Problem Stream

In principle, MSF’s idea that conditions must be turned into public problems to become relevant for the policy process should also remain valid in nondemocracies. Indicators, focusing events, and feedback are likely to be the main ways of learning about conditions that could be considered problems in autocracies, too. Nevertheless, there are at least two principal differences between democracies and nondemocratic systems that are relevant for the problem stream.

First, because autocratic systems tend to be centralised, the centre has to learn about a condition and needs to be convinced that the condition constitutes a problem in order for the problem stream to become ready for coupling. This may not be too different from at least some centralised democracies, though. What is probably different in autocracies, however, is the role of problem brokers. In democracies, all different kinds of people, from academics, interest group representatives, and journalists to political parties and civil servants, can be problem brokers, and they can use the media to make the public aware of the potential problem. Given the limits on or lack of media freedom in nondemocracies (Babayan et al. 2021; van den Dool 2022, p. 4), however, problem brokers are likely to direct their efforts to the leader because in many cases they cannot hope to get the leader’s attention via public debate on the problem.^1^ Hence, successful problem brokers in autocracies are very likely to come either from the people around the autocratic leader (Wu 2020, p. 247) or from the bureaucracy and public officials.

Moreover, the incentives for bureaucrats to report potential problems may differ depending on what the problem is. Feedback may occur less frequently in autocracies than in democracies, for example (cf. Babayan 2021, p. 3). If a policy does not work as expected, implementation has failed, or a focusing event has occurred that is due to the (in)actions of the bureaucracy or the inadequate policies of the autocrat, bureaucrats may have little incentive to report these conditions. They might fear that they will be punished for failures of government policies and prefer to sweep the issue under the rug—which might be a real option if media are strictly censored. In contrast, if a potential problem occurs that has nothing to do with previous government policy (a natural disaster, for example), bureaucrats might be more willing to report about the condition. Thus, we hypothesise the following:

H1: If a potential problem occurs that does not result from previous government policy, it is more likely that problem brokers use one or more of the following attention-generating mechanisms to engage in framing a condition as problematic: worsening indicators, harmful focusing events, and feedback regarding policies that do not work as expected.

Second, the decision-making centre, even if it learns about a potential problem, might choose to ignore the issue. The more strictly it controls the media, the better it will be able to deny the existence of any condition that might be considered a problem (for China, see van den Dool 2022, p. 4). An autocrat might not want to deal with a potential problem for a number of reasons. One is that the government will not be eager to declare its previous policy approach a failure if it can avoid it—and absent free media, it can avoid it.

Nonetheless, some autocratic leaders also delegate authority. To the extent that the autocrat has done so in a specific issue area, she or he might be willing to permit public debate on a potential problem in that policy field because she or he can deflect blame and make the institution or person to which authority has been delegated responsible for the problem at stake (Schuler 2020). Problem brokers anticipate an issue’s chances for consideration in public debate, which leads us to hypothesise the following:

H2: It is more likely that problem brokers engage in framing a condition as a problem if the condition falls into a portfolio that the autocratic leader has delegated.

Another and related reason for ignoring potential problems could be that the problem does not “fit” ideologically; i.e., the potential problem runs counter to ideological positions of the regime or questions its main policy concerns. For example, a dictator committed to neoliberal economic policy might not be willing to admit that inequality has become a problem. Similarly, an autocrat might want to ignore the severe consequences of a catastrophe to maintain regime autonomy, for instance, by avoiding having to accept international aid in exchange for the implementation of policies imposed by the international aid agency. Finally, autocrats may not have particularly strong incentives to deal with problems. Given the absence of competitive elections, they do not have to fear being voted out of office for their failure to respond to a potential problem. These considerations lead to the following hypothesis:

H3: It is more likely that problem brokers succeed in framing a condition as a problem if it does not run counter to the regime’s ideology or if it involves questions of autonomy.

These points lead us to expect that autocratic governments tend to respond less swiftly to problems and are more likely to ignore them for longer periods of time (see also Jones et al. 2019). The less free the media are, the more this pattern should prevail. It might have become more difficult to control the media even for autocrats in times of social networks and the internet, however. For example, Wu (2020, p. 243, 245) presents evidence that in some instances, traditional and new media were instrumental in bringing a problem to policy-makers’ attention in China (see also Ge et al. 2020, p. 4; He and Li 2021). Hence, to the extent that somewhat free media reporting is possible in an autocracy, the processes of problem recognition and definition could be more open. Under these conditions, problem brokers could come from outside the bureaucracy and the autocrat’s surroundings, too. Hypothesis 4 summarises our expectations regarding problem brokers:

H4: The more restricted media reporting is, the more likely it is that only individuals from the people around the autocratic leader, from the bureaucracy, and from the ranks of public officials act as problem brokers.

Moreover, under some circumstances, autocratic governments may respond much more rapidly to new problems. First, from an MSF perspective, Herweg et al. (2015, p. 437) have argued that a condition’s relevance for policy-makers in democracies is related to the extent the condition could affect reelection. Similarly, autocratic leaders could become willing to consider a condition a problem if it is relevant for regime stability (or survival of the ruling party; van den Dool 2022, p. 6). Moreover, selectorate theory (Bueno de Mesquita et al. 2003) would lead us to expect that if members of a regime’s winning coalition are hurt by the potential problem, the autocrat is more likely to attend to it. Hence, we hypothesise the following:

H5: The more politically relevant (in terms of regime stability or the autocrat’s political power) a condition is considered to be by the autocrat, the more likely she or he is to attend to that issue as a problem.

Second, as already implied by H3, autocratic leaders might be particularly attentive to issues that are strongly related to their ideology or their main policy concerns. For example, North Korean leaders probably have considered the slightest sign of military activity south of their border as a defence problem. Similarly, during the Cold War, the socialist autocracies in Eastern Europe were very sensitive to welfare state issues in order to substantiate their ideological claim to extraordinary social protection for their citizens (“workers’ paradise”). Hence, they even entered into competition with the West in terms of welfare state expenditure (Obinger and Schmitt 2011). The finding that regime competition played a role in this regard is particularly interesting from an MSF perspective because falling behind competitors has long been considered an important aspect of problem definition in MSF thinking (Kingdon 1984, p. 117). This argument is theoretical corroboration of H3, so we do not formulate a further hypothesis.

Summing up, the problem stream does seem to travel well to autocratic regimes. Also in these systems, policy-makers need to become aware of potential problems. Problem brokers are likely to come from the bureaucracy and the surroundings of the leader, while problem brokers from the outside play a less important role, depending on how restricted media freedom is. Problem brokers engage in framing conditions as problems if a relevant indicator changes, a harmful focusing event occurs, or feedback points to policy failure. There is likely a bias regarding the conditions the government attends to, first, because government failures stand a smaller chance of being reported by the bureaucracy and the media and hence feedback might be less important, and second because the leader will be highly attentive to avoid failure in issues that are ideologically important, while she or he might ignore many other potential problems.

### The Policy Stream and Policy Entrepreneurs

At least theoretically, one can expect that policy proposals are developed in policy communities in authoritarian regimes, too (cf. Zhu 2008 and Babayan et al. 2021 as examples). For example, a policy community consisting of a group of advisors, civil servants, and policy-makers, most of them trained at the University of Chicago (hence called “Chicago boys”), substantially shaped Chilean economic policy during the Pinochet years (Silva 1991; Kogut and Macpherson 2008). At the same time, it seems likely that policy communities in autocracies are small and exclusive, as the autocratic leader (or the leader’s government/party/junta) will probably have picked the experts. Hence, the policy community will include few (if any) people from outside government, the bureaucracy, or the ruling party. Yet in an early extension of the MSF, Zahariadis and Allen (1995), analysing British and German privatisation policies, already distinguished different kinds of policy communities. Hence, the policy communities we are likely to find in nondemocratic systems could resemble integrated policy communities, which are small, consensual, and allow only restricted access—just like the ones Zahariadis and Allen found in Germany—although in autocracies, of course, “there is only limited space to openly deliberate and mobilize support for policy proposals” (van den Dool 2022, p. 5).

Similarly, scholars have had no problem in applying the role of policy entrepreneur in autocratic systems: “There is no inbuilt necessity that policy entrepreneurs appear only in systems that have elections or a free media” (Hammond 2013, p. 121; see also Zhu 2008; Ge et al. 2020; Wu 2020). Because the policy entrepreneur comes from the policy community, according to the MSF, the same restrictions apply regarding the group of people who can act as policy entrepreneurs. That is to say, policy entrepreneurs are also very likely to come from inside the state apparatus or to be close to the decision-making centre. Depending on the specific autocracy, the military can also act as policy entrepreneur, as Obinger and Kovacevic (2016) show for education and social policy in the Habsburg empire. On the other hand, there is evidence from case studies on Belarus, Russia, and China that nongovernmental organisations (NGOs) can also act as policy entrepreneurs under certain conditions (Babayan et al. 2021; Bindman et al. 2019; He and Li 2021). Apart from that, their success is likely to be related to the same factors as for their counterparts in liberal democracies (see also van den Dool 2022, p. 6 for China), as summarised in the following hypothesis:

H6: Policy entrepreneurs are more likely to couple the streams successfully during an open policy window if they have more access to core policy-makers, are more persistent, and have good negotiating skills.

While policy communities can exist in nondemocracies, the criteria, which members of the policy community use to assess the suitability of a proposal—the so-called criteria for survival—could differ from the ones prevalent in democracies. Zhu (2008), for example, argues for the Chinese case that—in contrast to liberal democracies—politically acceptable but technically infeasible proposals stand a better chance of being successful in autocratic regimes than technically feasible proposals because the former are more likely to garner the government’s attention. It does not seem plausible, however, that such a pattern can be generalised for policy-making in autocracies. Even scholars of Chinese policy-making point out that Zhu’s (2008) argument is an “interesting contribution but of seemingly limited application” (Hammond 2013, p. 123).

Nonetheless, there are good reasons to expect that the relative importance of the different criteria for survival could differ between democracies and nondemocracies. Most studies of China, including the ones by Zhu (2008) and van den Dool (2022), show that ideology is of utmost importance for proposals to become viable policy alternatives (Liu and Jayakar 2012, p. 23). Clearly, a proposal that is not in line with the ideology of the Communist party does not stand a chance of getting adopted. More generally, anticipated approval of the current leader is of utmost importance in any nondemocratic setting. In a democracy, the members of a policy community might not necessarily eliminate a proposal from consideration only because the current government is unlikely to adopt it—the proposal’s time might come after the next election. In autocracies, a change of government is much less likely, which makes anticipated approval of the current leaders a sine qua non for further consideration of a proposal. This might not be too much of a restriction empirically, however, because the policy community in autocracies is usually close to the autocratic leader anyway, so it will likely come up with proposals that are in line with the leadership’s preferences (Bindman et al. 2019, but see Babayan et al. 2021).

Financial viability may also be an important issue for many proposals given the financial constraints of many autocracies (cf. Jafari et al. 2017, p. 407). At the same time, funding may not be particularly constraining for leaders’ pet projects—particularly because the population is unable to electorally punish the leader for wasting public money. For the same reason, technical feasibility might not be of prime importance either.

H7: If it is unlikely that a policy proposal will get the autocratic leader’s approval, the likelihood of gaining agenda status and thus being coupled decreases significantly.

H8: If policy proposals are of utmost importance for the autocratic leader but do not fulfil the criteria of financial viability and/or technical feasibility, they are still likely to gain agenda status and to be coupled successfully.

Taken together, we argue that the main concepts of the policy stream should also be applicable to nondemocracies. Policy communities are likely to be integrated, and policy entrepreneurs will mostly not come from outside the circles of the leader and her or his group. Regarding the criteria for survival, the leader’s approval is most probably the dominating criterion. Financial viability and technical feasibility might take a back seat instead, at least if projects of prime ideological importance or prestige for the leader are concerned.

### The Political Stream

As explained previously, the core elements of the political stream are governments and parliaments (and changes in their composition), interest groups, and the national mood. In applications of the MSF to political systems other than the U.S. presidential system that it was developed for, this stream was the one that needed the most adaptations (Herweg et al. 2018). It seems evident that the need to adjust the political stream should also apply to nondemocracies in which changes of government are rare (in contrast to changes in the composition of the political elite), interest groups cannot be established freely, and leaders have far fewer incentives to follow the national mood in the absence of competitive elections. Hence, the literature also attests a particular need to adjust the political stream (for example, Ge et al. 2020, p. 3; Liu and Jayakar 2012, p. 25). Therefore, the next question becomes whether the political stream can be adapted to the peculiarities of autocracies at all. We think the answer is yes.

It is clear that in autocratic systems, the leader is the dominating actor in the political stream. If the autocrat does not support policy change, it is unlikely to come about. The autocrat’s support may hinge upon her or his ideological preferences, the ambition to remain in office, the interests of the winning coalition, or the suggestions of the people close to her or him. Unlike in democracies, the term of office of autocratic leaders is not limited.^2^ Hence, changes of government (or leaders) should be much rarer in autocracies than in democracies. Nonetheless, if leaders change in an autocracy—infrequently as that may happen—that is likely to make a difference in the political stream.

While autocratic leaders clearly dominate the political stream, they will be unable to attend to all issues and develop preferences on all policies themselves. Hence, there are other actors (usually close to the leader) who can act as political entrepreneurs.^3^ It is likely that these actors bring certain ideas to the policy-making process and hence can make a difference. Consequently, changes in these positions may constitute a change of key personnel in Kingdon’s (1984, p. 160) sense.

At the same time, much of the politics in autocracies could play out inside the government machinery or the ruling party. Liu and Jayakar (2012, p. 23), for example, identify interministerial competition as driving the political stream in their case study on China (in a similar vein, see Gilli et al. 2018). This is not that different from policy-making in democracies, either; however. Kingdon (1984, p. 162–167), in his original contribution, also described “turf battles” as an important part of the political stream. Therefore, we hypothesise the following:

H9: Policy proposals that the autocratic leader supports have a better chance of gaining agenda status.

H10: In issue areas in which the autocratic leader has delegated authority, policy proposals that the responsible executive supports have a better chance of gaining agenda status.

At the same time, interest groups could be more relevant than a first glance at nondemocratic regimes might suggest. It is certainly correct that the establishment of interest groups is rarely free in autocratic regimes. While this is highly problematic from a normative point of view, it does not necessarily imply that the policy process is completely different. Remember also that in democracies not all interests have the same chances of affecting policy (Olson 1965). So what is relevant—in both democracies and nondemocracies—is the balance of support of existing interest groups. As long as relevant interest groups exist, they should also be accounted for in autocracies. This results in the following hypothesis:

H11: In systems in which interest groups are allowed to operate, policy proposals stand a better chance of gaining agenda status if the autocratic leader perceives that the balance of support among interest groups is in favour of the proposal.

Intuitively, one could argue that the national mood should be irrelevant in autocracies. In democracies, governments have to worry that they will be voted out of office if they ignore public opinion. As this mechanism does not exist in autocracies, policy-makers have no reason to consider public opinion (see Jones et al. 2019). At the same time, however, while reelection is usually not an issue in autocracies, regime stability is (Gandhi et al. 2020). Hence, at least for some highly salient issues, the national mood could be relevant, as empirical studies on China (Ge et al. 2020; Truex 2020; Wu 2020) and Vietnam (Schuler 2020) suggest. What is more, even the totalitarian regime in Nazi Germany regularly monitored public opinion (cf. Boberach 1984), which also fed into social policy considerations, for example (cf. Obinger et al. 2021, p. 409–10).

H12: If the national mood touches on salient issues pivotal for regime stability, these issues have a better chance of gaining agenda status.

Autocrats depend not only on the public’s loyalty but also on elites’ loyalty (Williamson and Magaloni 2020). Hence, coming back to selectorate theory (Bueno de Mesquita et al. 2003), one could argue that, at least regarding issues of moderate salience for the general public, it is the mood of the regime’s selectorate or winning coalition that matters. If the autocratic leader perceives that the members of her or his winning coalition or the selectorate more generally favour a specific policy change, she or he might be more willing to adopt it. Hence, the national mood might turn into a “selectorate’s mood” in autocracies. Therefore, we hypothesise the following:

H13: If the autocratic leader perceives that the members of her or his winning coalition or the selectorate more generally favour specific policy proposals, they have a better chance of gaining agenda status.

In conclusion, it does seem possible to adapt the political stream to autocratic systems. The autocrat clearly dominates that stream—but that is not so different from arguments about parliamentary systems, for example, for which Herweg et al. (2015, p. 439) maintain that governing parties are the most relevant actors. Nonetheless, other elements of the political stream remain important, too, because the dictator is unlikely to have clear policy preferences in many cases and does not have enough time and resources to attend to all issues. Hence, political entrepreneurs will focus on the leader (or the group of leaders) to win over their support. At the same time, there might be competition for the autocrat’s attention between various political entrepreneurs. What is more, depending on the specific autocratic system, even interest groups and the national or selectorate’s mood could become relevant for policy-making processes in autocracies.

### The Policy Window and Coupling the Streams

From an MSF perspective, policy windows open either in the problem stream or in the political stream. In democracies, problem windows are likely to open if a condition threatens the government’s reelection (Herweg et al. 2015, p. 437). A similar argument can be made for autocracies. If a potential problem becomes so dramatic that it threatens regime stability, that problem is likely to open a policy window. Indeed, Wu (2020) argues that some problems caused so much concern among the Chinese population that the Communist Party had to respond fast. Moreover, selectorate theory (Bueno de Mesquita et al. 2003) suggests that the autocrat’s survival also hinges on the winning coalition of the selectorate. Hence, a problem window is also likely to open if the dictator believes that a potential problem may hurt the members of the winning coalition and might lead them to seek a new leader. Hence, just as in democracies, the desire to remain in office might also incite autocratic leaders to consider some conditions to be problems that need to be dealt with. We hypothesise the following:

H14: The more a condition puts the autocratic leader’s position or regime stability at risk, the more likely it is to open a policy window in the problem stream.

Political windows, in contrast, open due to changes in the political stream. While changes of government (or leaders) are likely to be much rarer in autocracies than in democracies, if leaders change in an autocracy, that will very likely open a policy window. The incoming leader may act as a political entrepreneur and listen to other policy entrepreneurs than her or his predecessor did or have specific policy proposals herself that she or he may wish to see adopted. If that is the case, we are likely to see remarkable change. Examples include the Chinese economic reforms after Deng Xiaoping became (de facto) leader of the People’s Republic of China at the end of the 1970s and the policy changes that occurred under the heading of perestroika after Mikhail Gorbachev became General Secretary of the Communist party in the Soviet Union in 1985.

Other changes are more frequent in the political stream of autocracies. Examples include changes in the higher ranks of bureaucracies, new leading members of the party machinery, the appointment of a new minister, or the entry of a new member into the inner circle of the dictator. These changes may also open a policy window because these individuals may be open to new ideas, and policy entrepreneurs who did not have access to their predecessors might approach them to instil their policy proposals into the policy process (van den Dool 2022, p. 5–6). If the policy entrepreneurs are able to win the support of these officials, the latter can become political entrepreneurs and try to seek support from the autocrat for the proposal.

As outlined above, the national mood and what we termed the selectorate’s mood also play a role in nondemocracies’ political streams. Hence, if they change, a policy window opens.

These considerations lead to the following:

H15: The policy window opens in the political stream as a result of at least one of the following changes: change of the autocratic leader, changes in other leadership positions, changes in the national mood, or changes in the selectorate’s mood.

According to the MSF’s core hypothesis, agenda change becomes more likely if all streams are ready for coupling, a policy window opens, and a policy entrepreneur succeeds in coupling the streams. Transferring Kingdon’s (1984) reasoning to autocracies, policy entrepreneurs’ main task in problem windows is to find a solution that fits the problem on the agenda and gets the autocrat’s or responsible executive’s support. In contrast, if the policy window opens in the political stream, policy entrepreneurs’ main task is to find a problem with her or his favourite policy that the autocrat or executive responsible supports. Given the dominant role of the autocratic leader, it seems plausible that we also observe commissioning (Ackrill and Kay 2011) as a coupling strategy. With commissioning, a policy-maker, in this context an autocratic leader, selects the policy alternative that she or he deems appropriate for the policy window and, consequently, the policy entrepreneur (whose favourite policy happens to be the alternative the autocratic leader has selected), who then engages in advocating this alternative.

Because the analysis of prelegislative policy struggles in autocracies is still in its infancy (Gandhi et al. 2020), further research is required to specify the particularities of coupling activities in these regimes. However, building on Wu (2020), we can add another condition that influences a policy entrepreneur’s chances of coupling the streams successfully (apart from her or his access to the core policy-maker, persistence, and negotiating skills; see hypothesis 6). Wu (2020, p. 246) shows how opposition by the real estate industry and local governments was able to block a reform on Chinese urban demolition policy twice. Similarly, Ge et al. (2020) report an important role of interest groups in transportation policy in China. Therefore, we hypothesise the following:

H16: It is more likely that a policy entrepreneur will succeed in coupling the streams if important interest groups do not oppose the envisaged policy change.

### Explaining Decision-Making

So far, we have focused on adjusting the MSF to explain agenda-setting processes in autocracies. But the MSF is equipped to explain decision-making processes, too. However, as in democracies, institutions matter more during decision-making than during agenda-setting. Therefore, the organisation of the legislative process in autocracies varies with regime type (Williamson and Magahoni 2020) and also over time (Wilson and Woldense 2019). This variety renders different MSF explanations of policy change necessary. In the following, we briefly illustrate that expanding the MSF to decision-making might also be a worthwhile approach for studying autocracies.

Amendments of draft bills are surprisingly common in autocracies (Noble 2020). Chen et al. (2010, quoted in Ma and Lin 2012, p. 102) even argue that consensus building is particularly important in China “*because* of the absence of a democratic system and policy arena” (emphasis added). These findings challenge the idea that once an issue gets on the decision agenda in an autocracy, it most likely will be adopted (cf. Jones et al. 2019; Wu 2020). Rather, the question concerns when the legislature acts as a rubber stamp and simply formalises the decision already made by the autocrat (Jones 1984) and when (and why) it does not.

As in democracies, we distinguish two scenarios regarding policy change: Scenario one is policy adoption without (significant) amendments, and scenario two is policy adoption with amendments. In democracies, policy adoption without amendments is more likely if the draft proposal was supported by a parliamentary majority during agenda coupling and if that parliamentary majority does not depend on other actors’ consent to pass the draft (Herweg et al. 2015; Zohlnhöfer et al. 2016). Similary, in nondemocracies, policy adoption without amendments is more likely if the draft bill was backed by the entire political elite during decision coupling (Gandhi et al. 2020, p. 1364).

However, the scenario in which policy adoption occurs with amendments is the one in which applying MSF is most likely to reveal new insights into policy dynamics. In democracies, policy adoption with amendments is more likely if the initiator of a draft bill (i.e., a party or coalition) lacks internal cohesion or depends on the approval of other legislators or a second chamber due to institutional requirements (Herweg et al. 2015; Zohlnhöfer et al. 2016). In this case, MSF analyses focus on the question of how political entrepreneurs use package deals, concessions, and manipulation to put together the majority needed in parliament to pass the draft.

In nondemocracies, veto players do not trigger amendments, but key stakeholders within the ruling coalition do (Truex 2020). Thus, policy adoption with amendments is more likely if agenda-setting was characterised by disagreements within the ruling elite. Such disagreement implies that the draft bill allows for thinking about the same circumstances or phenomena in different ways, which is the defining feature of ambiguity (Feldman 1989). One might wonder why the ruling elite does not settle its disagreement before introducing a draft bill. As Noble (2020) points out, time pressures associated with the envisaged policy change is one key explanation for expanding disagreements into decision-making—which fits nicely with MSF’s assumption of policy-makers acting under time constraints.

How do political entrepreneurs use this ambiguity to negotiate amendments? To answer this question, we need to answer first who acts as political entrepreneurs. In contrast to democracies, in nondemocracies political entrepreneurs do not necessarily take over a draft bill from the policy entrepreneur because it is their favourite policy. According to recent research, political entrepreneurs in authoritarian legislatures “serve as proxy fighters” (Lü et al. 2020, p. 1380) for members of the ruling elite with different policy preferences. Put differently, members of the ruling elite task members of decision-making bodies to work on amending the proposal to make it more closely resemble their favourite policy (Noble 2020; Williamson and Magaloni 2020). Thus, decision coupling is the continuation of the disagreement within the ruling elite during agenda coupling.

Regarding political entrepreneurs’ coupling strategies, it seems plausible that they apply the same strategies as their democratic counterparts to change the draft proposal in a way that makes it (more) congruent with the preferences of the dissenting part of the ruling elite (i.e., package deals, concessions, and manipulation). Krol (2017), for instance, has documented that in Russia, amendments made to executive-introduced draft bills resulted from concessions to regime-loyal legislators.

However, since legislative politics in nondemocracies has only been researched incidentally (Gandhi et al. 2020), it is a yet unanswered research question under which circumstances members of decision-making bodies manage to strike package deals, negotiate concessions successfully, and use manipulation to change the proposal in the ruling elite’s sense. The MSF seems to be a promising candidate to answer this question. However, given the huge institutional differences between autocratic regimes, the findings might be specific for one regime type and their applicability to other regime types subject to further research.

## Conclusions

Is MSF being stretched too far by being applied to autocratic systems? The short answer is no. But we do acknowledge there are issues of conceptual stretching and a need for adaptation (Sartori 1970; Collier and Mahon 1993). As we mentioned above, many assumptions in democratic systems are valid in autocratic systems as well. However, some might need to be relaxed a little. In addition, some elements may also need to be differently conceptualised, such as the national mood in the political stream. In democratic systems, public opinion or a general “reading of the times” may play a role. In countries where no such luxuries exist, the main area of concern might not be the national mood but the selectorate’s or party mood. In other words, some elements may have to be combined or reconceptualised to fit the environment. Does this increase the risk of overstretching? As Sartori (1970) made clear many years ago, concepts need to be malleable, and they must be able to travel up the ladder of abstraction so as to remain true to their original intent but still apply to diverse environments. The implication is that the more widely a concept travels, the more abstract it needs to be, so some kind of balance is appropriate.

The biggest concern is not whether MSF travels well to autocratic regimes but whether there is enough information to discern what exactly is happening there. The lack of information, because it is either hidden, uncollected, or distorted, raises significant questions of efficacy. In opaque systems, it is easy to attribute superhuman resources or powers to those in charge, such as the Communist party in China. But does it have all the power it is charged with? Theory tells us that large public bureaucracies are often plagued by corruption and misallocation of resources (Wilson 1991; Acemoglu and Verdier 2000). The reason is simple: There is little information by design and by accident and even less interest in collecting it for accountability reasons. Therefore, combining elements into variables may also be a matter of necessity, for now.

We have shown that MSF elements are specified at a level abstract enough to be able to travel to autocratic systems. Whether democratic or autocratic, timing, ambiguity, and the need to build coalitions of support are still essential ingredients of policy-making. Perhaps concepts of fairness and justice need to be adjusted, but autocratic policy-makers are just as likely to have problematic preferences and suffer from time constraints as are democratic politicians, bills are still debated, and there is sometimes disagreement, opposition, and frustration with lack of participation. All are perfectly normal elements of the policy process.

## Footnotes

1 This is not to deny that there are empirical instances in which problem brokers who were not close to the leader were able to generate media attention (mostly via social media) and draw the attention of the dictator to an issue, as we discuss later.

2 Note that we do not consider elections in nondemocracies in our discussion of the political stream because they have a different role in authoritarian regimes and differ tremendously across countries, levels, and time (for a summary, see Gandhi and Lust-Okar 2009).

3 The role of the political entrepreneur (“an actor who holds an elected leadership position”) needs to be defined slightly more broadly, namely as an actor who holds a leadership position—election cannot be a prerequisite for that position in autocracies.
